# Nurses’ Use of mHealth Apps for Chronic Conditions: Cross-Sectional Survey

**DOI:** 10.2196/57668

**Published:** 2024-05-29

**Authors:** Wa'ed Shiyab, Kaye Rolls, Caleb Ferguson, Elizabeth Halcomb

**Affiliations:** 1 School of Nursing Faculty of Science, Medicine and Health University of Wollongong Wollongong Australia

**Keywords:** mHealth apps, adoption, smartphone, chronic conditions, nursing, technology, chronic, nurse, nurses, mHealth, mobile health, app, apps, use, cross-sectional, survey, surveys, questionnaire, questionnaires, mobile phone

## Abstract

**Background:**

Mobile health (mHealth) is increasingly used to support public health practice, as it has positive benefits such as enhancing self-efficacy and facilitating chronic disease management. Yet, relatively few studies have explored the use of mHealth apps among nurses, despite their important role in caring for patients with and at risk of chronic conditions.

**Objective:**

The aim of the study is to explore nurses’ use of mHealth apps to support adults with or at risk of chronic conditions and understand the factors that influence technology adoption.

**Methods:**

A web-based cross-sectional survey was conducted between September 2022 and January 2023. The survey was shared via social media and professional nursing organizations to Australian nurses caring for adults with or at risk of chronic conditions.

**Results:**

A total of 158 responses were included in the analysis. More than two-thirds (n=108, 68.4%) of respondents reported that they personally used at least 1 mHealth app. Over half (n=83, 52.5% to n=108, 68.4%) reported they use mHealth apps at least a few times a month for clinical purposes. Logistic regression demonstrated that performance expectancy (*P*=.04), facilitating condition (*P*=.05), and personal use of mHealth apps (*P*=.05) were significantly associated with mHealth app recommendation. In contrast, effort expectancy (*P*=.09) and social influence (*P*=.46) did not have a significant influence on whether respondents recommended mHealth apps to patients. The inability to identify the quality of mHealth apps and the lack of access to mobile devices or internet were the most common barriers to mHealth app recommendation.

**Conclusions:**

While nurses use mHealth apps personally, there is potential to increase their clinical application. Given the challenges reported in appraising and assessing mHealth apps, app regulation and upskilling nurses will help to integrate mHealth apps into usual patient care.

## Introduction

Chronic conditions account for 74% of all deaths globally [1]. Approximately 46% to 53% of adults in high-income countries have at least 1 chronic condition [2,3]. The high prevalence of chronic conditions contributes significantly to premature morbidity and mortality as well as poor quality of life [1,4]. Chronic conditions are also linked to high health care costs and resource consumption [5]. Self-management is a key strategy shown to improve outcomes and quality of life [6]. The growth of technology has changed how people can be supported to self-manage their chronic conditions [6,7]. Mobile health (mHealth) apps are an example of such technology.

In recent years, the use of mHealth apps has increased widely, driven by a global exponential increase in internet access, mobile phone ownership, and other smart technologies, such as wearables such as smartwatches and fitness trackers. It is estimated that in 2021, more than 350,000 mHealth apps were available from the Apple Store and Google Play [8]. Almost half of these apps were for chronic conditions, such as mental health, diabetes, and cardiovascular disease [8]. Apps offer a wide range of functionalities, including medication calculation, symptom monitoring and tracking, health data collection and monitoring, and access to health information [9]. Such functionality has widespread application and use in a range of chronic conditions.

Previous studies have suggested that mHealth apps could improve self-efficacy and adoption of healthy behaviors and empower individuals to engage more strongly in self-management [10,11]. The easy accessibility of mHealth apps makes them a viable adjunct to traditional health care by facilitating communication between patients and health care providers, especially when face-to-face visits are inaccessible [12]. The popularity of these apps was evident through the significant increase in downloads of exercise, mental health, and blood pressure management mHealth apps during the COVID-19 pandemic [8].

Despite the potential value of mHealth apps to improve patient care and health outcomes, the often slow progress of trials, along with the rapid changes in innovations, features, and functions of mHealth apps, make it difficult to keep the evidence up to date with the technology [13]. Additionally, mHealth apps need to be embedded in clinical practice to fully realize their efficacy [14]. While the role of mHealth apps in usual clinical practice is not well understood [15], a likely key to their maximum efficacy is uptake and engagement by the health care professionals providing care [16].

The unified theory of acceptance and use of technology (UTAUT) was used to guide this study in terms of understanding clinician engagement. The UTAUT is based on the assumption that there are 3 direct determinants of intention to use (performance expectancy, effort expectancy, and social influence) and 2 direct determinants of usage behavior (intention to use and facilitating condition) [17,18]. In total, 4 factors (gender, age, experience, and voluntariness of use) act as moderators and key features of the UTAUT [17,18]. These key principles guided the survey design.

Previous studies have explored the adoption of mHealth apps among health professionals such as doctors, pharmacists, and dieticians [9,19,20]. Despite nursing being the largest single health care profession globally [21] and being involved in delivering many interventions to support self-management, relatively few studies have explored the use of mHealth apps among nurses [19]. Previous studies of nurses have reported that the use of mHealth apps is relatively low, despite many nurses identifying that they are useful [22]. de Jong et al [23] report that nurses mainly use mHealth apps for checking medication information, reviewing laboratory tests, and communicating with other health care professionals and patients. However, being distracted by using their smartphone, concerns that patients might have negative feelings regarding device use, privacy, data security concerns, and lack of organizational support are perceived by nurses as key barriers to mHealth app use [23]. Gaining insight into nurses’ engagement with the rapid advances of mHealth apps will help empower them to get the maximum benefit of such advances. This has the potential to enhance patient care, strengthen self-management support, and optimize health outcomes.

## Methods

### Objectives

This paper seeks to explore Australian nurses’ use of mHealth apps to support adults with or at risk of chronic conditions and understand the factors that influence technology adoption.

### Design

A web-based cross-sectional survey was conducted between September 2022 and January 2023 as the first phase of a concurrent mixed methods study. The second phase consisted of semistructured interviews with a subgroup of survey respondents. Given the depth of the data and the different foci, these interview data are reported separately.

### Respondents

Diploma-prepared enrolled nurses, baccalaureate-prepared registered nurses, and master’s-prepared nurse practitioners who identified as caring for adults with or at risk of chronic conditions across Australia were eligible to participate. Respondents were recruited through social media (Facebook and Twitter) and professional nursing organizations, such as the Australian College of Nursing, the Australasian Cardiovascular Nursing College, and the Australian Primary Health Care Nurses Association. Social media posts provided brief study information and the survey link. Follow-up and reminder posts were made at regular intervals [24]. Professional organizations advertised the study either via their email lists, social media pages, newsletters, or electronic communications.

### Data Collection

The survey was web-based and delivered via REDCap (Research Electronic Data Capture; Vanderbilt University) [25]. The survey tool was developed by the research team based on expert knowledge and previous studies [9,20,22]. It consisted of 3 sections. The first section collected demographic and professional characteristics, including employment setting, age, gender, professional designation, work experience, location, education, clinical role, and specialty area.

The second section explored the use of mHealth apps for professional and clinical purposes. This section was based on previous surveys [9,20,22] and explored mHealth apps that are used by nurses for personal purposes, how respondents used mHealth apps for professional purposes, and whether respondents were asked to or recommended mHealth apps to patients.

The last section comprised a 38-item scale devised by Lim et al [20] (used with author permission). The first 19 items addressed factors influencing the adoption of mHealth apps in clinical work and the other 19 items addressed factors that affect the prescription of mHealth apps for patients. Each item is rated on a 5-point Likert scale, from 1=strongly disagree to 5=strongly agree. These items are based on the 4 constructs of the UTAUT, namely, performance expectancy, effort expectancy, social influence, and facilitating conditions [17] (Table S1 in Multimedia Appendix 1).

### Validity and Reliability

The survey was assessed for face validity by a convenience sample of 5 nurses before dissemination. These nurses had expertise in survey design and the use of technology in health care and chronic conditions. The feedback they provided was used to amend the wording and format of the tool.

Lim et al [20] demonstrated acceptable reliability for the 38-item scale, with Cronbach α for each construct being 0.67-0.89. In this study, the Cronbach α for each construct was between 0.69 and 0.93, which indicates good reliability (Table S1 in Multimedia Appendix 1).

### Ethical Considerations

The study was approved by the Human Research Ethics Committee of the University of Wollongong (approval 2022/202) in July 2022. Respondents were provided with an information sheet at the commencement of the survey and asked to check a box if they consented to participate. Participants were free to withdraw participation at any time during the survey, which can be done by ceasing the survey. However, once the survey was submitted the participants were not able to withdraw their responses as the data generated was deidentified. Any contact details that were provided in the survey were separated prior to analysis. Two AUD $100 vouchers were allocated to randomly selected participants who responded to the survey and were willing to be included in the prize draw. The value of the prize vouchers was sufficient to incentivize participation by compensating respondents for their time without being an inducement.

### Data Analysis

The data were exported from REDCap into SPSS (version 28; IBM Corp) for analysis. Records were considered incomplete if more than half of the survey was not completed. Incomplete records were removed before analysis. Where less than half of the data were missing, the response was included, and the data were categorized as missing in the analysis.

Descriptive statistics were used to summarize the demographic and professional characteristics [26]. Age data were grouped based on standardized generational groups [27]. Logistic regression, using factors from the literature, was used to identify the factors that influence the adoption of mHealth apps. *P*<.05 was considered to be statistically significant.

## Results

### Respondents’ Characteristics

Although 207 nurses responded to the survey, 49 (23.7%) surveys were incomplete and so were excluded. Therefore, 158 (76.3%) responses were included in the analysis. Most respondents were female (n=134, 84.8%), had completed a graduate diploma and above (n=112, 70.8%), and worked in a metropolitan area (n=100, 63.3%; Table 1). Slightly less than half of respondents (n=76, 48.1%) were from Generation X (43-58 years).

**Table 1:** Personal and professional characteristics (n=158).

Attribute			Values
Age (years), mean (SD)			46.7 (10.7)
**Age group, n (%)**			
	Millennials (26-42 years)	50 (31.6)	
	Generation X (43-58 years)	76 (48.1)	
	Boomer (59-73 years)	20 (12.7)	
	Missing data	12 (7.6)	
**Education, n (%)**			
	Diploma or bachelor’s degree	46 (29.1)	
	Graduate certificate or diploma	49 (31)	
	Master’s degree	56 (35.4)	
	PhD degree	7 (4.4)	
**State, n (%)**			
	New South Wales	92 (58.2)	
	Victoria	26 (16.5)	
	Queensland	14 (8.9)	
	Western Australia	9 (5.7)	
	South Australia	5 (3.2)	
	Australian Capital Territory	5 (3.2)	
	Northern Territory	4 (2.5)	
	Tasmania	2 (1.3)	
	Missing data	1 (0.6)	
**Work location, n (%)**			
	Metropolitan or urban	100 (63.3)	
	Rural or regional	45 (28.5)	
	Remote area	5 (3.2)	
	Missing data	8 (5.1)	
**Professional designation, n (%)**			
	Registered nurse	80 (50.6)	
	Clinical nurse consultant	25 (15.8)	
	Clinical nurse specialist	17 (10.8)	
	Nurse practitioner	11 (7)	
	Clinical nurse educator	7 (4.4)	
	Enrolled nurse	6 (3.8)	
	Nurse manager	5 (3.2)	
	Multiple roles	14 (8.8)	
**Chronic condition, n (%)**			
	Cardiovascular disease	55 (34.8)	
	Diabetes mellitus	30 (19)	
	Multiple chronic conditions	18 (11.4)	
	Respiratory disease	16 (10.1)	
	Neurologic disease	13 (8.2)	
	Mental health condition	10 (6.3)	
	Missing data	1 (0.6)	
	Others	15 (9.5)	
**Employment setting, n (%)**			
	Acute care setting	51 (31)	
	General practice	38 (23.4)	
	Community health	32 (19.6)	
	Outpatient specialist service	28 (17.1)	
	Others	9 (5.7)	

Of the 158 respondents, half (n=80, 50.6%) were registered nurses, with a further 33.6% (n=53) employed in advanced practice roles (eg, clinical nurse consultant, clinical nurse specialist, and nurse practitioner). Slightly less than a third (n=50, 31.7%) of respondents had worked in nursing for 20 years or less. Some (n=55, 34.8%) respondents primarily cared for patients with cardiovascular diseases, and 41.1% (n=65) provided chronic episodic care. Only 31% (n=51) of respondents worked in an acute hospital setting.

### Personal mHealth App Use

Of the 158 respondents, most (n=108, 68.4%) reported that they personally used at least 1 mHealth app. The most popular mHealth apps used by respondents were physical activity trackers (n=77, 48.8%), mindfulness and meditation apps (n=45, 28.5%), symptom trackers (n=37, 23.5%), and diet trackers (n=34, 21.5%).

Personal use of mHealth apps was significantly associated with both age and gender (Table 2). Generation X (43-58 years) used mHealth apps 3 times more than boomers (59-73 years; *P*=.04), while millennials (22-42 years) used mHealth apps 5 times more than boomers (*P*=.008). Female respondents were twice as likely to use mHealth apps compared with male respondents (*P*=.04). Education and work location were not significantly associated with personal use of mHealth apps (*P*>.05).

**Table 2:** Predictors of mHealth^a^ app personal use.

Factors		OR^b^ (95% CI)	*P* value
**Age group**			
	Millennials (22-42 years)	4.926 (1.524-15.920)	.01^c^
	Generation X (43-58 years)	3.125 (1.069-9.135)	.04^c^
	Boomers (59-73 years)	1 (—^d^)	—
**Sex**			
	Female	1 (—)	—
	Male	.341 (.122-.956)	.04^c^
**Education**			
	Undergraduate	1 (—)	—
	Postgraduate	.998 (.417-2.389)	.99
**Work location**			
	Remote area	1 (—)	—
	Rural or regional	.434 (.039-4.803)	.49
	Metropolitan or urban	.402 (.038-4.83)	.45

^a^mHealth: mobile health.

^b^OR: odds ratio.

^c^Significant values.

^d^Reference group.

### mHealth App Use in Practice

Of the 158 respondents, only 2.5% (n=4) reported not having internet access, and 7% (n=11) of respondents reported that internet access is not provided by their employer. For clinical purposes, over half of the respondents reported they use mHealth apps at least a few times a month to communicate with other health professionals or colleagues (n=108, 68.4%); get information about medications or calculate dosages (n=99, 62.7%); access clinical guidelines, protocols, or reference sources (n=93, 58.9%); and interact with electronic medical records (n=83, 52.5%; Table 3).

**Table 3:** Clinical mHealth^a^ app uses (n=158).

How often do you use a mHealth app	Never, n (%)	Less than once a month, n (%)	A few times a month, n (%)	Few times a week, n (%)	At least once a day, n (%)
To access a scientific journal	38 (24.1)	45 (28.5)	40 (25.3)	23 (14.6)	12 (7.6)
To access clinical guidelines, protocols, or reference sources	31 (19.6)	34 (21.5)	46 (29.1)	29 (18.4)	18 (11.4)
To get information about medications or calculate dosages	25 (15.8)	34 (21.5)	35 (22.2)	37 (23.4)	27 (17.1)
To interact with electronic medical records	61 (38.6)	14 (8.9)	10 (6.3)	16 (10.1)	57 (36.1)
To communicate with other health professionals or colleagues	33 (20.9)	17 (10.8)	23 (14.6)	34 (21.5)	51 (32.3)
To communicate with patients or their families	82 (51.9)	17 (10.8)	16 (10.1)	15 (9.5)	28 (17.7)
To book a shift or manage your roster	70 (44.3)	16 (10.1)	25 (15.8)	28 (17.7)	19 (12)

^a^mHealth: mobile health.

Respondents’ perceptions toward using mHealth apps in clinical practice were variable. Approximately one-third of the 158 respondents agreed that performing tasks on mHealth apps is easy (n=56, 35.4%), that mHealth apps facilitate clinical decision-making (n=51, 32.3%), and that they can control the use of mHealth apps (n=51, 32.3%). These items reflect effort expectancy, performance expectancy, and facilitating conditions, respectively. The social influence of using mHealth apps was generally low (n=10, 6.3% to n=40, 25.3%). Only 10.2% (n=16) of respondents thought that mHealth apps could improve the quality of care, and 13.9% (n=22) agreed that information from mHealth apps is up-to-date (see Table S1 in Multimedia Appendix 2 for additional details).

### mHealth App Recommendation

Of the 158 respondents, slightly fewer than half (n=74, 46.8%) reported that they recommend mHealth apps to patients at least once a month. Similarly, 64 (40.5%) respondents reported that they were asked for recommendations for mHealth apps at least once a month. Respondents reported not recommending mHealth apps for various reasons (Table 4). The most reported barriers were not being sure how to identify the quality of mHealth apps (n=65, 41.1%) and the lack of access to mobile devices or internet (n=53, 33.5%). The least commonly reported barriers were not being within their scope of practice (n=16, 11.1%) and privacy concerns (n=26, 16.5%).

**Table 4:** Barriers to mHealth^a^ app recommendation (n=158).

Barriers	Values, n (%)
Not sure how to identify the quality of mHealth apps	65 (41.1)
Lack of access to mobile device or internet	53 (33.5)
Not confident in recommending mHealth apps	45 (28.5)
Unsure if mHealth apps improve health outcomes	42 (26.6)
Concern about the cost of apps	42 (26.6)
Patients are not interested	38 (24)
Concerns about liability if there are issues with using apps	31 (19.6)
I do not think patients can use apps	30 (19)
Never crossed my mind	27 (17.1)
Concerns about patient privacy	26 (16.5)
Not in my scope of practice	16 (11.1)
Other barriers	5 (3.2)

^a^mHealth: mobile health.

Factors that influence mHealth app recommendations were assessed based on the 4 constructs of the UTAUT. Slightly less than half of 158 respondents felt that mHealth apps could encourage patients to gain more health knowledge (n=77, 48.8%), and more than a third believed that mHealth apps improve chronic disease management (n=63, 39.9%) and patients’ health (n=59, 37.5%). These 3 items all reflect performance expectancy. The social influence items were perceived as the lowest, with only 10.8% (n=17) of respondents reporting that patients adhered to the mHealth apps that they recommended to them, 12% (n=19) of respondents thought that the organization has a plan to implement mHealth app use for patients, and 13.3% (n=21) of respondents believed that the organization supports mHealth app recommendations (see Table S2 in Multimedia Appendix 2 for additional details).

Logistic regression demonstrated that performance expectancy (*P*=.04), facilitating condition (*P*=.05), and personal use of mHealth apps (*P*=.05) were significantly associated with mHealth app recommendation (Table 5). In contrast, effort expectancy (*P*=.09) and social influence (*P*=.46) did not have a significant influence (*P*>.05) on whether respondents recommended mHealth apps to patients.

**Table 5:** Predictors of mHealth^a^ app recommendation.

Factors	OR^b^ (95% CI)	*P* value
Personal use of mHealth app	2.668 (1.002-7.106)	.05^c^
Performance expectancy	2.384 (1.038-5.476)	.04^c^
Effort expectancy	.328 (.092-1.171)	.09
Social influence	1.553 (.481-5.014)	.46
Facilitating condition	3.743 (1.000-14.006)	.05^c^

^a^mHealth: mobile health.

^b^OR: odds ratio.

^c^Significant values.

## Discussion

### Principal Findings

This paper has explored the current use of mHealth apps among Australian nurses and the factors that influence technology adoption. Understanding the current situation regarding nurses’ mHealth app use, preferences, and experiences given the recent rapid advancements in mHealth apps will inform future interventions, practices, and policies to support self-management for those living with chronic conditions. Strategies to empower nurses to maximize the benefit of mHealth apps will likely positively impact patient care and health outcomes [28].

Findings revealed that respondents’ personal use of mHealth apps was similar to other health care providers, which ranged from 60% to 76% [29,30]. This highlights the widespread use and familiarity of health care providers with mHealth apps. It is noteworthy that, in this study, personal use of mHealth apps was found to be a significant predictor of their recommendation to patients. Other studies have also found that health professionals’ personal use of mHealth apps significantly impacted their recommendations to patients [29,30]. The relationship between personal use and recommendation of mHealth apps suggests that it may be possible to leverage the pre-existing familiarity of health care providers with mHealth apps through workforce development [30]. This includes promoting the digital capabilities of nurses as a part of continuous professional development to adapt to a rapidly changing digital world [31]. In addition, encouraging knowledge-sharing and peer-to-peer learning can be a strategy to build digital literacy [32].

Despite the high personal use of mHealth apps, this study found that the inability to discern reliable apps and a lack of confidence in recommendations were the top barriers to mHealth app recommendations. Similar challenges were reported in previous studies, which reported unawareness of effective apps and sources to access them [9,22]. These barriers highlight the importance of mHealth app regulation, including involving the nurses in the whole process of mHealth app development [33], as well as the establishment of a rigorous framework for appraising mHealth apps, which could help nurses identify and differentiate high-quality apps for patient use [34]. On an individual level, Ferguson and Jackson [35] discussed criteria to evaluate app quality, and recently, more work has been done by the Australian Digital Health Agency to create a framework to help in the assessment of the quality and safety of mHealth apps [34]. This challenge is likely not confined to Australia. In their study of mHealth app regulation in 9 countries, Essén et al [36] found that all these countries have some initiatives, and despite the fact that the United Kingdom, Belgium, and Germany advanced in developing frameworks for app appraisal, they still struggle with implementation. Although the rapid developments in technology challenge policy makers and researchers, concerted efforts to create a unified and validated framework for app appraisal are still needed. Moreover, to maximize the benefit of such frameworks, nurses need to be provided with appropriate training to implement and use these frameworks in their practice [22].

Beyond the quality assessment frameworks to be used by individual clinicians, a further strategy to support app recommendation in clinical practice is a library that embraces safe and reliable apps and provides critical appraisals [9,22,37]. Regular reassessment of the quality of included apps is needed to keep such libraries up-to-date [34]. These measures could improve health care providers’ confidence in recommending apps, which ultimately will reflect on the quality of care provided to patients [9,22].

Other predictors of mHealth app recommendation in this study were performance expectancy and facilitating conditions. Consistent with Lim et al [20], performance expectancy was found in this study to be significantly associated with app recommendation. Performance expectancy refers to the extent to which people believe that using technology will provide a gain in job performance [17]. Nurses’ beliefs about the importance of mHealth apps in the management of patient conditions could positively influence them to recommend these apps to patients. Based on these findings, providing nurses with reliable evidence about the efficacy of mHealth apps will likely increase their rate of recommendation to patients [20]. Facilitating conditions, which encompass self-control over using apps, data security, time, and app affordability to patients [17], were also found to be significantly associated with app recommendation. This is a significant finding for mHealth app developers to give more attention to the app design. Secure, reliable, and trusted apps, with free or minimal cost, are more likely to be recommended to patients [20].

### Limitations

This is one of the few studies that has explored the adoption of mHealth apps among nurses and the factors that are associated with such adoption. However, there are some limitations to this study. Given the inherent low response rate in survey research and survey fatigue, the sample size was modest. However, to improve the response rate, evidence-based strategies were followed for recruitment [24]. Despite the modest size, the sample did provide a spread of respondents across demographic groups and clinical settings. Another limitation is the absence of a validated tool to explore the adoption of mHealth apps, so the previous literature acted as a guide for the development of the survey tool. Finally, a bias may exist, as the sample might not be representative of the broader population of nurses. As in most survey research, the respondents might be more interested in the survey topic than those who declined to respond.

### Conclusions

Overall, this research demonstrated that many nurses use mHealth apps personally, which increases the likelihood of adopting them in clinical practice and fosters patients’ autonomy to self-manage their chronic conditions. However, given the large number of mHealth apps and the lack of regulation of these apps, nurses face challenges in integrating these apps into routine patient care. Targeting the barriers that nurses face would promote the integration of mHealth apps and harness their potential for the benefit of health care providers and patients. Nurses’ involvement in any proposed solutions is essential.

## Appendix

Multimedia Appendix 1 Unified theory of acceptance and use of technology (UTAUT) constructs reliability.

Multimedia Appendix 2 Factors influencing mobile health (mHealth) app recommendation and use in clinical practice.

## Abbreviations

mHealth: mobile health
REDCap: Research Electronic Data Capture
UTAUT: unified theory of acceptance and use of technology
