# Supplementary material for: Nurses’ Use of mHealth Apps for Chronic Conditions: Cross-Sectional Survey
Source: JMIR Nurs. 2024 May 29;7:e57668. doi: 10.2196/57668 (PMC11170041; doi:10.2196/57668)
Supplement: Multimedia Appendix 1 [file nursing_v7i1e57668_app1.docx]

TableS1: UTAUT constructs reliability

| **Items about the use of mobile health apps to support nurses’ clinical work** | | | |
| --- | --- | --- | --- |
| **Constructs** | **Items** | **Mean±SD** | **Cronbach’s Alpha** |
| **Performance expectancy** | mHealth apps increase my productivity. | 3.21±0.86 | 0.878 |
|  | mHealth apps facilitate my clinical decision-making. |  |  |
|  | mHealth apps reduce medication errors. |  |  |
|  | mHealth apps help me to improve patient care. |  |  |
| **Effort expectancy** | It is easy to perform tasks on the mHealth apps. | 3.04±0.79 | 0.768 |
|  | Interaction with the mHealth apps is easy. |  |  |
|  | I prefer to use a mobile device to find the information I need compared to a computer. |  |  |
| **Social influence** | My colleagues use mHealth apps. | 2.75±0.63 | 0.781 |
|  | My colleagues recommend that I use more mHealth apps. |  |  |
|  | Patients find it intrusive when I use mHealth apps during a clinic session. |  |  |
|  | Patients believe that mHealth apps can improve the quality of care. |  |  |
|  | The clinic organization supports the use of mHealth apps in the clinic. |  |  |
|  | The clinic organization has a strategic plan to implement mHealth app usage in the clinic. |  |  |
| **Facilitating condition** | Information from mHealth apps is up to date. | 3.10±0.57 | 0.693 |
|  | My use of mHealth apps is entirely under my control. |  |  |
|  | I have time to use mHealth apps in my clinic. |  |  |
|  | mHealth apps are affordable. |  |  |
|  | I have concerns about data protection when using mHealth apps. |  |  |
|  | mHealth apps are generally valid and accurate. |  |  |
| **Items about the use of mobile health apps that recommended to patients by nurses** | | | |
| **Constructs** | **Items** | **Mean±SD** | **Cronbach’s Alpha** |
| **Performance expectancy** | mHealth apps improve patient health. | 3.47±0.81 | 0.931 |
|  | mHealth apps improve patient chronic disease management. |  |  |
|  | mHealth apps encourage patients to gain more health knowledge. |  |  |
| **Effort expectancy** | It is easy to recommend mHealth apps to patients. | 3.02±0.69 | 0.789 |
|  | Patients can use mHealth apps relatively easily. |  |  |
|  | The information in patient mHealth apps is easy to understand. |  |  |
| **Social influence** | My colleagues recommended mHealth apps to their patients. | 2.82±0.62 | 0.861 |
|  | My colleagues recommended that I should recommend mHealth apps to patients. |  |  |
|  | Patients like it when I recommend them to use mHealth apps. |  |  |
|  | Patients give good feedback after I recommend them mHealth apps. |  |  |
|  | Patients adhere to the mHealth app that I recommend. |  |  |
|  | The clinic organization supports the recommendation of mHealth apps to patients. |  |  |
|  | The clinic organization has a strategic plan to implement mHealth app usage for patients. |  |  |
|  | Technical assistance for mHealth technologies is available in my clinic. |  |  |
| **Facilitating condition** | My recommendation of mHealth apps is entirely under my control. | 3.11±0.65 | 0.822 |
|  | I have enough time to recommend mHealth apps to my patients. |  |  |
|  | mHealth apps are affordable for my patients. |  |  |
|  | The data protection of patient mHealth apps is reasonable enough for me to recommend them to my patients. |  |  |
|  | Patient mHealth apps are generally valid. |  |  |
