# Supplementary material for: Nurses’ Use of mHealth Apps for Chronic Conditions: Cross-Sectional Survey
Source: JMIR Nurs. 2024 May 29;7:e57668. doi: 10.2196/57668 (PMC11170041; doi:10.2196/57668)
Supplement: Multimedia Appendix 2 [file nursing_v7i1e57668_app2.docx]

TableS1: Factor influence mHealth app use in clinical practice

| Statements | | **Strongly disagree**  **n(%)** | | **Disagree**  **n(%)** | | **Neutral**  **n(%)** | | | **Agree**  **n(%)** | | | **Strongly agree**  **n(%)** | | |
| --- | --- | --- | --- | --- | --- | --- | --- | --- | --- | --- | --- | --- | --- | --- |
|  |  | n | % | n | % | | n | % | | n | % | | n | % |
| 1. | mHealth apps increase my productivity. | 7 | 4.4 | 17 | 10.8 | | 71 | 44.9 | | 24 | 15.2 | | 11 | 7 |
| 2. | mHealth apps facilitate my clinical decision making. | 9 | 5.7 | 16 | 10.1 | | 54 | 34.2 | | 34 | 21.5 | | 17 | 10.8 |
| 3. | mHealth apps reduce medication errors. | 9 | 5.7 | 10 | 6.3 | | 71 | 44.9 | | 29 | 18.4 | | 11 | 7 |
| 4. | mHealth apps help me to improve patient care. | 6 | 3.8 | 6 | 3.8 | | 81 | 51.3 | | 28 | 17.7 | | 9 | 5.7 |
| 5. | It is easy to perform tasks on the mHealth apps. | 9 | 5.7 | 11 | 7 | | 54 | 34.2 | | 43 | 27.2 | | 13 | 8.2 |
| 6. | Interaction with the mHealth apps is easy. | 6 | 3.8 | 15 | 9.5 | | 71 | 44.9 | | 31 | 19.6 | | 7 | 4.4 |
| 7. | I prefer to use a mobile device to find information I need compared to a computer. | 5 | 3.2 | 20 | 12.7 | | 68 | 43 | | 31 | 19.6 | | 6 | 3.8 |
| 8. | My colleagues use mHealth apps. | 19 | 12 | 25 | 15.8 | | 46 | 29.1 | | 31 | 19.6 | | 9 | 5.7 |
| 9. | My colleagues recommend that I use more mHealth apps. | 13 | 8.2 | 26 | 16.5 | | 57 | 36.1 | | 28 | 17.7 | | 6 | 3.8 |
| 10. | Patients find it intrusive when I use mHealth apps during a clinic session. | 27 | 17.1 | 38 | 24.1 | | 55 | 34.8 | | 9 | 5.7 | | 1 | 0.6 |
| 11. | Patients believe that mHealth apps can improve quality of care. | 6 | 3.8 | 26 | 16.5 | | 82 | 51.9 | | 14 | 8.9 | | 2 | 1.3 |
| 12. | The clinic organisation supports the use of mHealth apps in the clinic. | 4 | 2.5 | 17 | 10.8 | | 80 | 50.6 | | 26 | 16.5 | | 3 | 1.9 |
| 13. | The clinic organisation has a strategic plan to implement mHealth apps usage in the clinic. | 18 | 11.4 | 24 | 15.2 | | 60 | 38 | | 24 | 15.2 | | 4 | 2.5 |
| 14. | Information from mHealth apps is up to date. | 27 | 17.1 | 31 | 19.6 | | 50 | 31.6 | | 18 | 11.4 | | 4 | 2.5 |
| 15. | My use of mHealth apps is entirely under my control. | 9 | 5.7 | 25 | 15.8 | | 45 | 28.5 | | 35 | 22.2 | | 16 | 10.1 |
| 16. | I have time to use mHealth apps in my clinic. | 14 | 8.9 | 33 | 20.9 | | 51 | 32.3 | | 28 | 17.7 | | 4 | 2.5 |
| 17. | mHealth apps are affordable. | 5 | 3.2 | 12 | 7.6 | | 82 | 51.9 | | 26 | 16.5 | | 5 | 3.2 |
| 18. | I have concerns about data protection when using mHealth apps. | 7 | 4.4 | 22 | 13.9 | | 57 | 36.1 | | 37 | 23.4 | | 7 | 4.4 |
| 19. | mHealth apps are generally valid and accurate. | 5 | 3.2 | 7 | 4.4 | | 81 | 51.3 | | 33 | 20.9 | | 4 | 2.5 |

TableS2: Factors influence mHealth app recommendation

| Statements | | **Strongly disagree**  **n(%)** | | **Disagree**  **n(%)** | | **Neutral**  **n(%)** | | **Agree**  **n(%)** | | **Strongly agree**  **n(%)** | |
| --- | --- | --- | --- | --- | --- | --- | --- | --- | --- | --- | --- |
|  |  | n | % | n | % | n | % | n | % | n | % |
| 1. | mHealth apps improve patient health. | 5 | 3.2 | 7 | 4.4 | 55 | 38.4 | 52 | 32.9 | 7 | 4.4 |
| 2. | mHealth apps improve patient chronic disease management. | 6 | 3.8 | 6 | 3.8 | 50 | 31.6 | 55 | 34.8 | 8 | 5.1 |
| 3. | mHealth apps encourage patients to gain more health knowledge. | 5 | 3.2 | 5 | 3.2 | 38 | 24.1 | 66 | 41.8 | 11 | 7.0 |
| 4. | It is easy to recommend mHealth apps to patients. | 8 | 5.1 | 30 | 19.0 | 51 | 32.3 | 28 | 17.7 | 7 | 4.4 |
| 5. | Patients can use mHealth apps relatively easily. | 6 | 3.8 | 27 | 17.1 | 63 | 39.9 | 28 | 17.7 | 2 | 1.3 |
| 6. | My colleagues recommended mHealth apps to their patients. | 11 | 7 | 28 | 17.7 | 55 | 34.8 | 27 | 17.1 | 3 | 1.9 |
| 7. | My colleagues recommended that I should recommend mHealth apps to patients. | 18 | 11.4 | 35 | 22.2 | 50 | 31.6 | 21 | 13.3 | 1 | 0.6 |
| 8. | Patients like it when I recommend them to use mHealth apps. | 5 | 3.2 | 11 | 7 | 73 | 46.2 | 34 | 21.5 | 2 | 1.3 |
| 9. | Patients give good feedback after I recommend them mHealth apps. | 5 | 3.2 | 10 | 6.3 | 78 | 49.4 | 30 | 19 | 2 | 1.3 |
| 10. | Patients adhere to the mHealth app that I recommend. | 6 | 3.8 | 13 | 8.2 | 89 | 56.3 | 15 | 9.5 | 2 | 1.3 |
| 11. | The clinic organization supports the recommendation of mHealth apps to patients. | 10 | 6.3 | 34 | 21.5 | 60 | 38 | 19 | 12 | 2 | 1.3 |
| 12. | The clinic organization has a strategic plan to implement mHealth apps usage for patients. | 17 | 10.8 | 34 | 21.5 | 53 | 33.5 | 18 | 11.4 | 1 | 0.6 |
| 13. | Technical assistance for mHealth technologies is available in my clinic. | 21 | 13.3 | 32 | 20.3 | 47 | 29.7 | 22 | 13.9 | 3 | 1.9 |
| 14. | My recommendation of mHealth apps is entirely under my control. | 6 | 3.8 | 18 | 11.4 | 46 | 29.1 | 45 | 28.5 | 10 | 6.3 |
| 15. | The information in patient mHealth apps is easy to understand. | 3 | 1.9 | 13 | 8.2 | 77 | 48.7 | 31 | 19.6 | 0 | 0 |
| 16. | I have enough time to recommend mHealth apps to my patients. | 8 | 5.1 | 36 | 22.8 | 42 | 26.6 | 35 | 22.2 | 4 | 2.5 |
| 17. | mHealth apps are affordable for my patients. | 4 | 2.5 | 18 | 11.4 | 69 | 43.7 | 32 | 20.3 | 2 | 1.3 |
| 18. | The data protection of patient mHealth apps is reasonable enough for me to recommend them to my patients. | 5 | 3.2 | 14 | 8.9 | 72 | 45.6 | 28 | 17.7 | 3 | 1.9 |
| 19. | Patient mHealth apps are generally valid. | 3 | 1.9 | 9 | 5.7 | 75 | 47.5 | 33 | 20.9 | 4 | 2.5 |
